# Using the concept of preperitoneal membrane anatomy in total extraperitoneal prosthesis: a preliminary report

**DOI:** 10.3389/fsurg.2023.1119788

**Published:** 2023-06-08

**Authors:** Suqiong Lin, Anran Hu, Huabin Zheng, Jinbo Fu, Penghao Kuang, Xiaoquan Hong, Rongliang Qiu, Yilong Fu

**Affiliations:** ^1^ Department of General Surgery, Zhongshan Hospital, Xiamen University, Xiamen, China; ^2^ Department of General Surgery, Second Hospital of Sanming City, Sanming, China; ^3^ Department of General Surgery, General Hospital of Changtai District, Zhangzhou, China; ^4^ The Third Clinical Medical College of Fujian Medical University, Fujian Medical University, Fuzhou, China

**Keywords:** inguinal hernia, laparoscopic, total extraperitoneal prosthesis (TEP), membrane anatomy, hernia

## Abstract

**Purpose:**

Total extraperitoneal prosthesis (TEP) is one of the most commonly used laparoscopic inguinal hernia repair procedures. This work aims to report the application of membrane anatomy to TEP and its value in intraoperative space expansion.

**Methods:**

The clinical data of 105 patients, from January 2018 to May 2020, with inguinal hernia who were treated with TEP (58 patients in the General Department of the Second Hospital of Sanming City, Fujian Province, and 47 patients in the General Department of the Zhongshan Hospital Affiliated to Xiamen University) were retrospectively analyzed.

**Results:**

All surgeries were successfully completed under the guidance of the concept of preperitoneal membrane anatomy. The operation time was 27.5 ± 9.0 min, blood loss was 5.2 ± 0.8 ml, and the peritoneum was damaged in six cases. The postoperative hospital stay was 1.5 ± 0.6 days, and five cases of postoperative seroma occurred, all self-absorbed. During the follow-up period of 7–59 months, there was no case of chronic pain and recurrence.

**Conclusion:**

The membrane anatomy at the correct level is the premise of a bloodless operation to expand the space while protecting adjacent tissues and organs to avoid complications.

## 1. Introduction

Total extraperitoneal prosthesis (TEP) is currently one of the most commonly used laparoscopic inguinal hernia repair procedures (1). Owing to the complex anatomical structure of the preperitoneal space, the narrow intraoperative operating space, peritoneum damage, and bleeding, TEP is difficult, and the learning curve is long (2). In 2010, Japanese scholars put forward the concept of “membrane anatomy”, which is now widely used in colorectal surgery (3). Membrane anatomy is a new anatomical theoretical framework based on the structure and function of the mesentery and mesangial bed. The theory of membranous anatomy is generalized to mesangial anatomy and mesangial bed anatomy (4). The mesentery refers to the fascia and serosa surrounding the internal organs, and the mesangial bed is the structure to which the mesentery is attached. Membrane anatomy includes plane surgery, fascia understanding, and mesorectal anatomy. It also involves concepts such as “membrane block”, “channel”, and “structure with a mesenteric bed” (5). More and more scholars in the field of hernia surgery pay attention to how to apply the theory of membrane anatomy to TEP (6). In this study, the concept of membrane anatomy was applied to TEP, and its application value in intraoperative space expansion was discussed.

## 2. Materials and methods

### 2.1. Patients

The clinical data of 105 patients, from January 2018 to May 2020, with inguinal hernia who were treated with TEP (58 patients in the General Department of the Second Hospital of Sanming City, Fujian Province, and 47 patients in the General Department of the Zhongshan Hospital Affiliated to Xiamen University) were retrospectively analyzed. The patients were all men and had an average age of 59 (32–80) years old. The median BMI was 22.5 (17.5–33.8) kg/m^2^. There were 95 cases of unilateral hernia, including 60 cases of indirect hernia, 33 cases of direct hernia, and 2 cases of compound hernia, and 10 cases of bilateral hernia, including 6 cases of indirect hernia and 4 cases of direct hernia. Each operation was recorded synchronously during the operation to standardize the operation process and ensure the consistency of the quality of the operation. All patients and their families fully understood the surgical method and signed the informed consent. This study was approved by the hospital ethics committee (No. IRB202004-202007-06).

### 2.2. Surgical procedures

**Figure 1:**
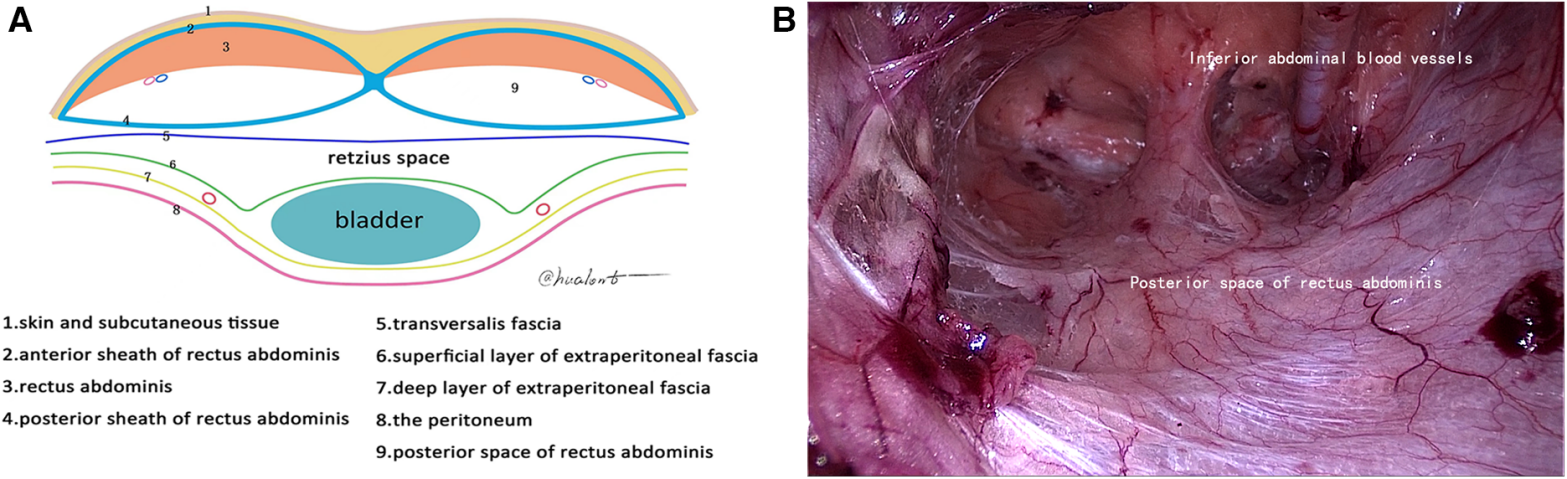
First reference plane. (**A**) Schematic diagram of the arcuate line; (**B**) posterior space of rectus abdominis.

**Figure 2:**
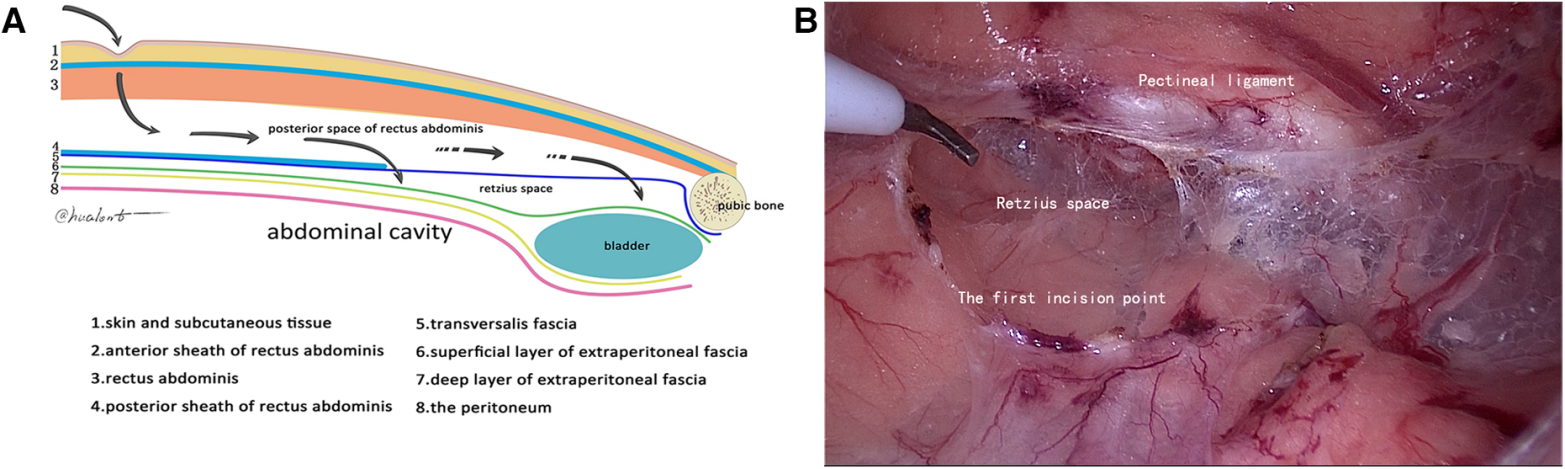
Second reference plane (the first incision point). (**A**) Schematic diagram of the first incision point; (**B**) cut the transversalis fascia 1 cm above the pectineal ligament.

**Figure 3:**
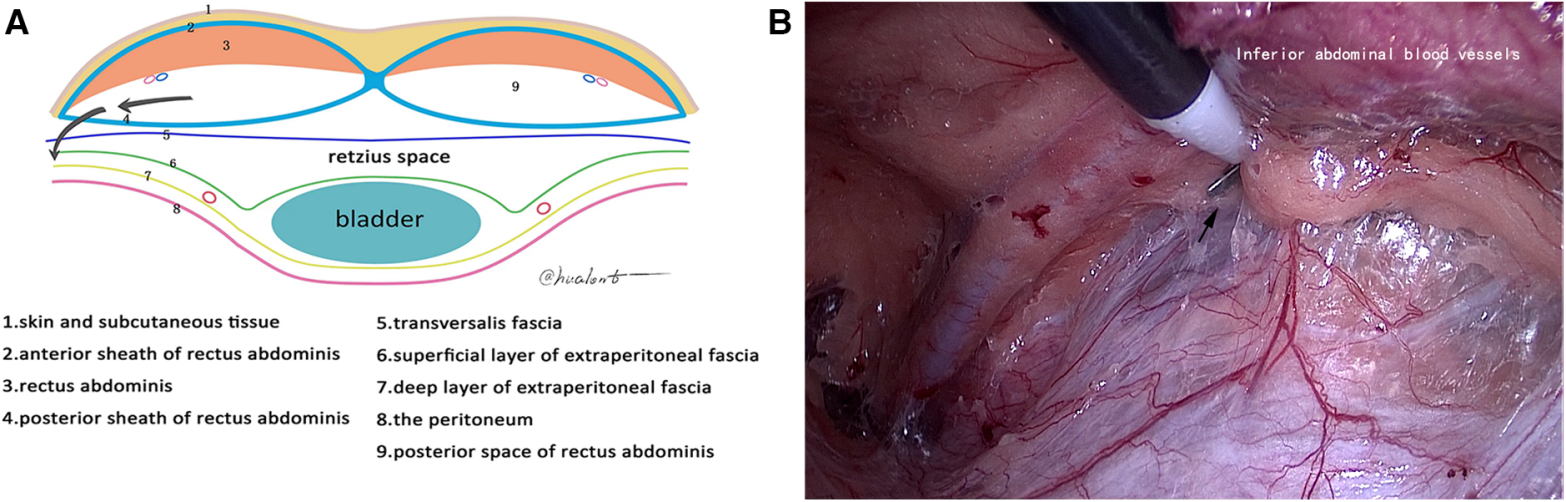
Second incision point. (**A**) Schematic diagram of the second incision point; (**B**) second incision point (black arrow).

**Figure 4:**
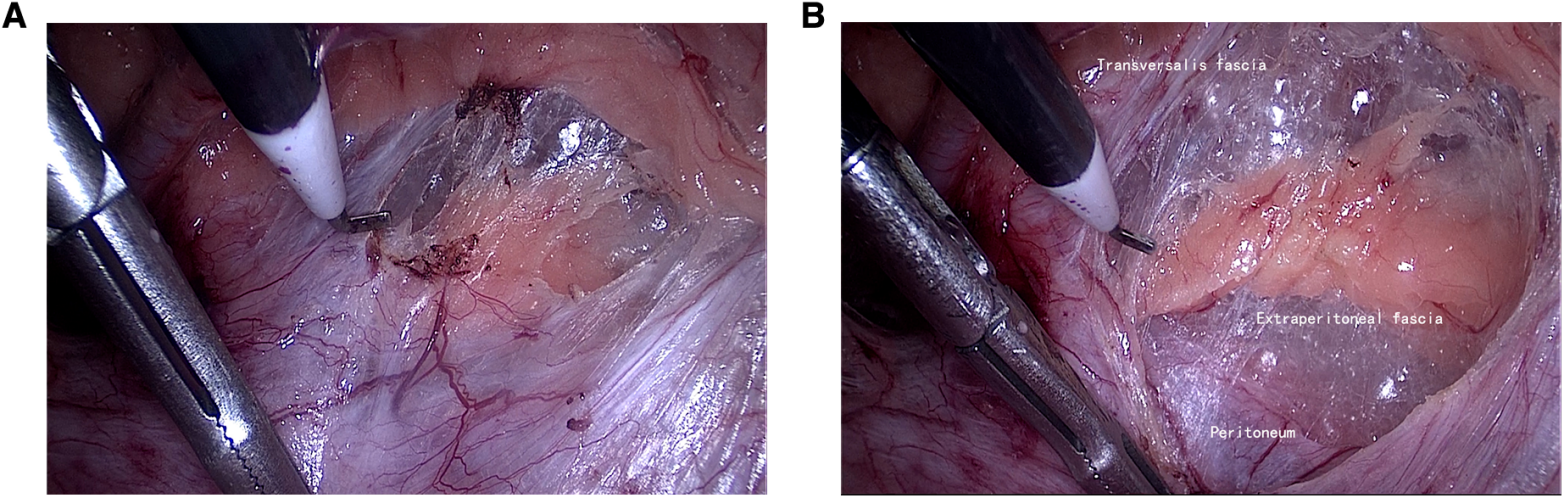
Third reference plane. (**A**) Cut the transversalis fascia (black arrow indicates the posterior sheath of the rectus abdominis); (**B**) enter the Bogros space.

Under general anesthesia with endotracheal intubation, the patient was placed in a supine position, a longitudinal incision was made through the lower border of the umbilicus, the anterior sheath of the rectus abdominis was cut under direct vision, and a 10-mm trocar was inserted to connect to the pneumoperitoneum and pushed along the front of the posterior sheath of the rectus abdominis to the lower border of the arcuate line by mirror pushing. The 5-mm trocar was placed at each trisection point of the line connecting the umbilicus and the midline of the pubic symphysis as an operating hole. We continued to dissociate the affected side with the posterior sheath of the rectus abdominis as the first reference plane, coagulate the small blood vessels that supply rectus abdominis, reach the attachment point of the posterior sheath of rectus abdominis laterally, and reach the lower edge of the arcuate line (Figure 1). Then, we used the transversalis fascia as the second reference plane to separate the pectineal ligament and always stayed at the expansion level of the anterior space of the transversalis fascia (separate in the same space). During this process, it was found that the transversalis fascia was attached to the pectineal ligament, and the transversalis fascia was incised approximately 1 cm away from the pectineal ligament and entered the Retzius space (the first incision point) to expand the Retzius space and fully expose the pubic tubercle and the pectineal ligament (Figure 2). To transition from the Retzius space to the Bogros space, the four-layer membranous structure (the second entry point) needs to be incised, and the posterior sheath should be properly incised from the lower edge of the arcuate line close to the attachment point of the posterior sheath of the rectus abdominis to expose the transversalis fascia. We dissected outward and found that the transversalis fascia attached to the iliopubic tract outward and downward (Figure 3). The transversalis fascia was incised to expose the superficial and deep layers of the extraperitoneal fascia, and the extraperitoneal fascia was incised to reach the peritoneum and enter the Bogros space (Figure 4). With the peritoneum as the reference plane, the “peeling” method was mainly used to expand the Bogros space and protect the lateral femoral cutaneous nerve and genitofemoral nerve. For direct hernias, the hernia sac is peeled off and brought back directly into the abdominal cavity. For indirect hernias, it is necessary to open the internal fascia of the spermatic cord to find the hernia sac close to the wall of the hernia sac to separate the hernia sac from the spermatic cord tissue; if the hernia sac is densely adhered or the scrotal hernia is huge, the proximal end can be ligated and the distal end can be left alone. The vas deferens were deperitoneized to the level of the atresia umbilical artery. A 10 cm × 15 cm polypropylene mesh was placed through the umbilical hole and placed flat and without curl, covering the entire musculopubic foramen. The hernia sac was lifted against the mesh, and the pneumoperitoneum was released under direct vision.

## 3. Results

All surgeries were successfully completed under the guidance of the concept of preperitoneal membrane anatomy. The operation time was 27.5 ± 9.0 min, the blood loss was 5.2 ± 0.8 ml, and the peritoneum was damaged in six cases. The postoperative hospital stay was 1.5 ± 0.6 days, and five cases of postoperative seroma occurred, all of which were self-absorbed. During the follow-up period of 7–59 months, there was no case of chronic pain and recurrence.

## 4. Discussion

In the field of hernia surgery, many domestic and foreign studies have elaborated on the membrane anatomy of the inguinal region, but there is a lack of unified understanding. Based on the theory of membrane anatomy proposed by Jianping (7), we combined our clinical experience to explore the membrane anatomy of the inguinal region under laparoscopy and gained a further understanding of the attachment points and layers of the membrane structure of each layer in the inguinal region: in the expansion of preperitoneal space guided by membrane anatomy, the surgical key points of “a gap, two entry points, and three reference planes” and the operation concept of “peeling” are put forward, which is helpful for the promotion and implementation of TEP.

### 4.1. A gap

Since Cooper proposed the concept of the transversalis fascia, the morphology and layering of the transversalis fascia have been controversial. Most scholars believe that the transversalis fascia is divided into superficial and deep layers; the superficial layer is denser and fused with the transversus aponeurosis, and the deep layer is a membranous structure, or if it is underdeveloped, the inferior epigastric vessels run between the two layers of the fascia. However, from the perspective of embryonic development, the theory of one layer of the transversalis fascia is more reasonable (8); that is, the deep layer of the transversalis fascia mentioned above is a layer of acellular fascia structure, surrounded by muscles and bones. In the groin region, its superficial surface is the transversus aponeurosis, and its deep membrane structure is the preperitoneal fascia. We agree with the view that the transversalis fascia is a single layer. During TEP, when the preperitoneal space was freed to the lower edge of the arcuate line, it was found that the transversalis fascia was close to the rear sheath of the rectus abdominis muscle, the medial was attached to the pectineal ligament, and the lateral was descended to become a part of the iliopubic tract. In current clinical practice, when the preperitoneal space is expended, the retro rectus abdominis space is dissociated first. Then, the transversalis fascia is incised at the lower edge of the arcuate line and transitions to the Retzius space for expansion. In this way, dissociation in different anatomical spaces is likely to form multiple layers and spaces after the gasification of CO_2_, which makes the surgeon unable to correctly grasp the surgical layer, too superficial to damage the blood vessels under the abdominal wall and too deep to damage the bladder. In this study, based on the observation of the layers and attachment points of the transversalis fascia, it is proposed that the transversalis fascia should not be incised at the lower edge of the arcuate line, and the integrity of the transverse fascia should be maintained. Continue to dissect the posterior space of the rectus abdominis in front of the transversalis fascia, reach the level of the pectineal ligament, and then enter the Retzius space, that is, the viewpoint of “a gap” (Figure 2A). A wider preperitoneal space can be obtained, and the surgical layer can be avoided from being too deep or too shallow so that the transition to the rear Retzius space and the lateral Bogros space is more in line with the principle of laparoscopic surgery from near to far.

### 4.2. Two entry points

From the perspective of hernia surgery, it is more reasonable to understand that the Retzius space is located between the transversalis fascia and the superficial layer of the preperitoneal fascia and that the Bogros space is located between the deep layer of the preperitoneal fascia and the peritoneum. Transitioning from the posterior space of the rectus abdominis to these two spaces is critical during TEP. This study proposes two fixed entry points from the posterior space of the rectus abdominis into these two spaces according to the attachment point of the membrane in the inguinal region. The former can avoid tearing the attachment site of the fascia and reduce the risk of rupture and bleeding of small blood vessels on the pectineal ligament. The latter allows safe access to the operating space with the peritoneum as the reference plane for Bogros space expansion.

### 4.3. Three reference planes

During the expansion of the preperitoneal space, the posterior sheath of the rectus abdominis, the transversalis fascia, and the peritoneum were used as reference surfaces successively, which were the reference points for the entire space expansion process and the key to integrating the concept of membrane anatomy into the TEP operation. In this study, the initial blunt extension of the rectus sheath was performed under direct vision to ensure the safe placement of the first trocar and the supply of small blood vessels (generally 2–3) from the inferior epigastric vessel to the rectus sheath was pretreated. Be sure to expand the space against the posterior sheath of the rectus abdominis to its insertion point. This kind of separation can clearly reveal the “yellow-white junction line”, i.e., the posterior sheath surface is white and the yellow fat layer is the tissue surrounding the inferior epigastric vessels (separation plane), so asto avoid damage to the inferior epigastric vessels due to too shallow separation and obtain a better field of vision. The dissection process from the inferior border of the arcuate line to the pectineal ligament takes the transversalis fascia as the reference plane (Figure 2). Owing to the different thicknesses of the transversalis fascia, some thin patients are often directly entered into the Retzius space due to the damage of the transversalis fascia. Therefore, the surgeon must use gentle movements, mainly peeling, to ensure the integrity of the transverse fascia as much as possible. Clinically, we have also observed that the transversalis fascia of young patients is thicker and tougher than that of elderly patients. Bogros space expansion involves the “pain triangle”, and protecting its superficial fascia is particularly important. Therefore, at the second entry point, the transversalis fascia cannot be cut to expand the space, and the peritoneal layer must be reached, and the Bogros space should be expanded using this as a reference plane (Figure 4). During the process, there will inevitably be small blood vessels that supply the peritoneum through the preperitoneal fascia. After cutting the small blood vessels, the “rivet” between the peritoneal fascia and the peritoneum will be cut off, and then the Bogros space will be separated by blunt dissection. This way, the genitofemoral nerve and lateral femoral cutaneous nerve deep into the fascia can be better protected.

In our opinion, the space expansion based on membrane anatomy in TEP should ensure the integrity of the transversalis fascia as much as possible, protect the bladder on the inside, and protect the nerves in the pain triangle on the outside. During the operation, fine anatomy and stripping-based surgical strategies were adopted to reduce the risk of intraoperative bleeding and thermal injury and finally obtain a large enough preperitoneal space to ensure that the mesh is placed flat and in place.

However, there are some limitations, such as a better standardization correlating technique, a longer observation time, a larger cohort, and more robust patient applied data. The long-term result needs to be verified by further research and follow-up.

## 5. Conclusion

In conclusion, for inguinal hernia surgery, only by mastering the preperitoneal membrane anatomy can one truly understand laparoscopic total extraperitoneal hernia repair. Membrane dissection at the correct level is the prerequisite for bloodless operation to expand the space while protecting adjacent tissues and organs to avoid complications.

## Data Availability

The original contributions presented in the study are included in the article, further inquiries can be directed to the corresponding author.
